# Supplementary material for: Measurement of the cosmic optical background using the long range reconnaissance imager on New Horizons
Source: Nat Commun. 2017 Apr 11;8:15003. doi: 10.1038/ncomms15003 (PMC5394269; doi:10.1038/ncomms15003)
Supplement: Supplementary Information — Supplementary Figures, Supplementary Tables and Supplementary References. [file ncomms15003-s1.pdf]

Supplementary Information

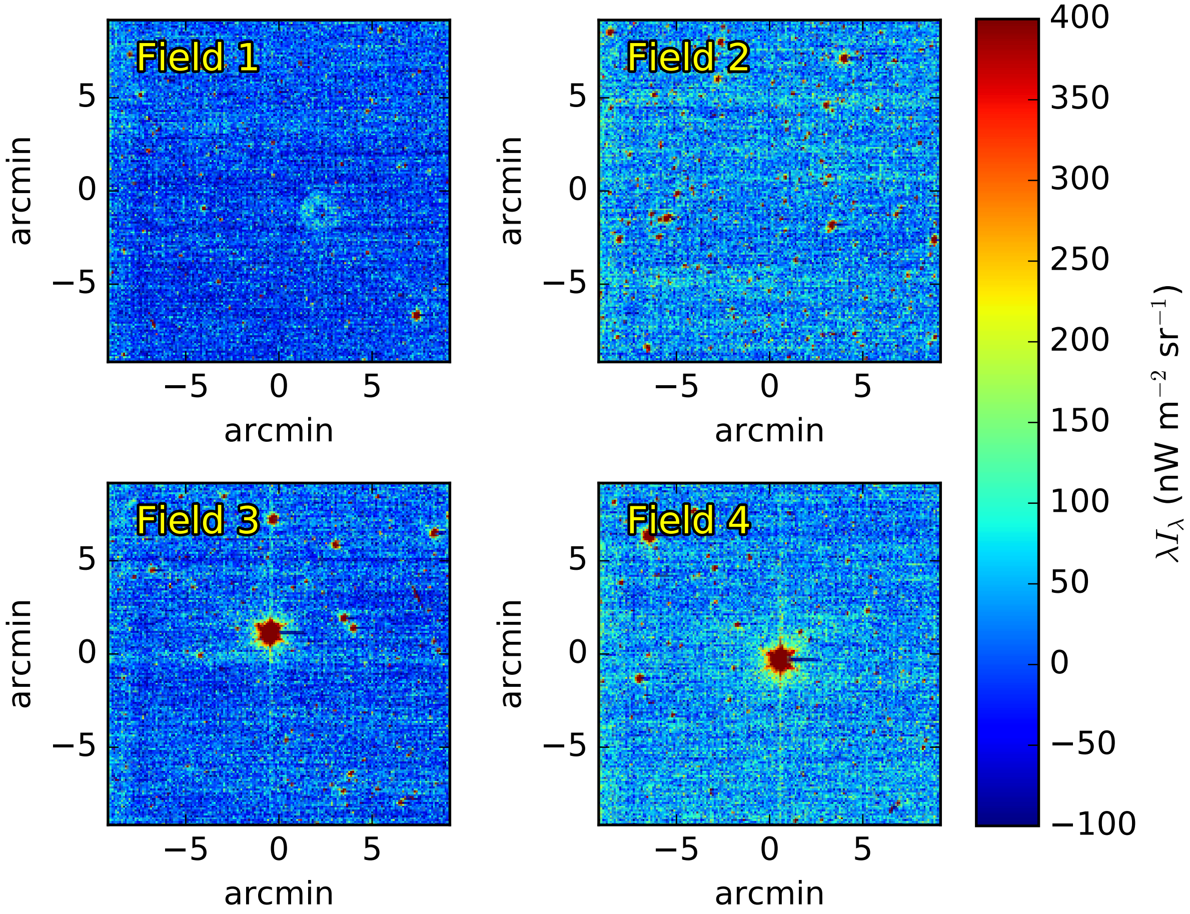

Supplementary Figure 1: **Reduced images of the four science fields used in this investigation, calibrated to surface brightness units.** Each panel shows one 10 s integration for each of the fields taken in rebinned 256×256 mode. Stars and Neptune (the large source in fields 3 and 4) are clearly visible. Field 1 exhibits optical ghosting from reflections in the field-flattening lens group. All of these structures are masked in later processing step to allow us to calculate the mean surface brightness away from known sources.

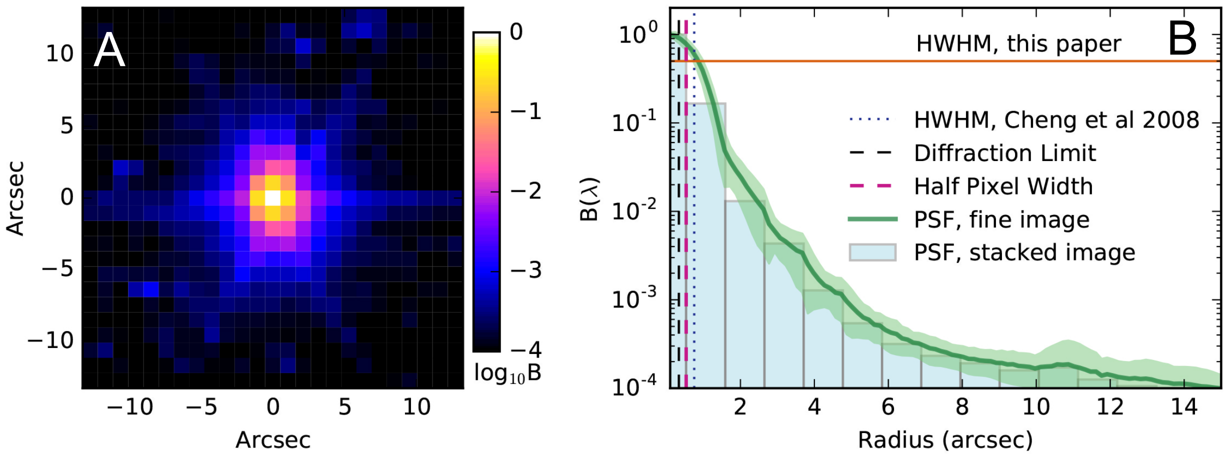

Supplementary Figure 2: **The point spread function (PSF) used for source masking in this work.** The 2-D PSF shown in panel A is produced by stacking star images on a fine pixel grid, and then averaging back into larger pixels[1]. Panel B is the annular average of the stacked PSF in the left panel (light blue columns), in comparison to the PSF of the original, fine stacked image (bold solid green line). The shaded region shows the standard deviation of the fine PSF in annuli. Also shown are the diffraction limit for the LORRI telescope (thin dashed line), the half pixel width (thick dashed line), the half width at half maximum (HWHM) of our PSF (marked by the crossing point of the horizontal solid line and the fine PSF), and the HWHM from previous determinations[2–4]. The HWHM of our PSF measurement is consistent with the previous determinations. Any asymmetry of the PSF at low levels is likely due to a small pointing drift, but does not affect our analysis.

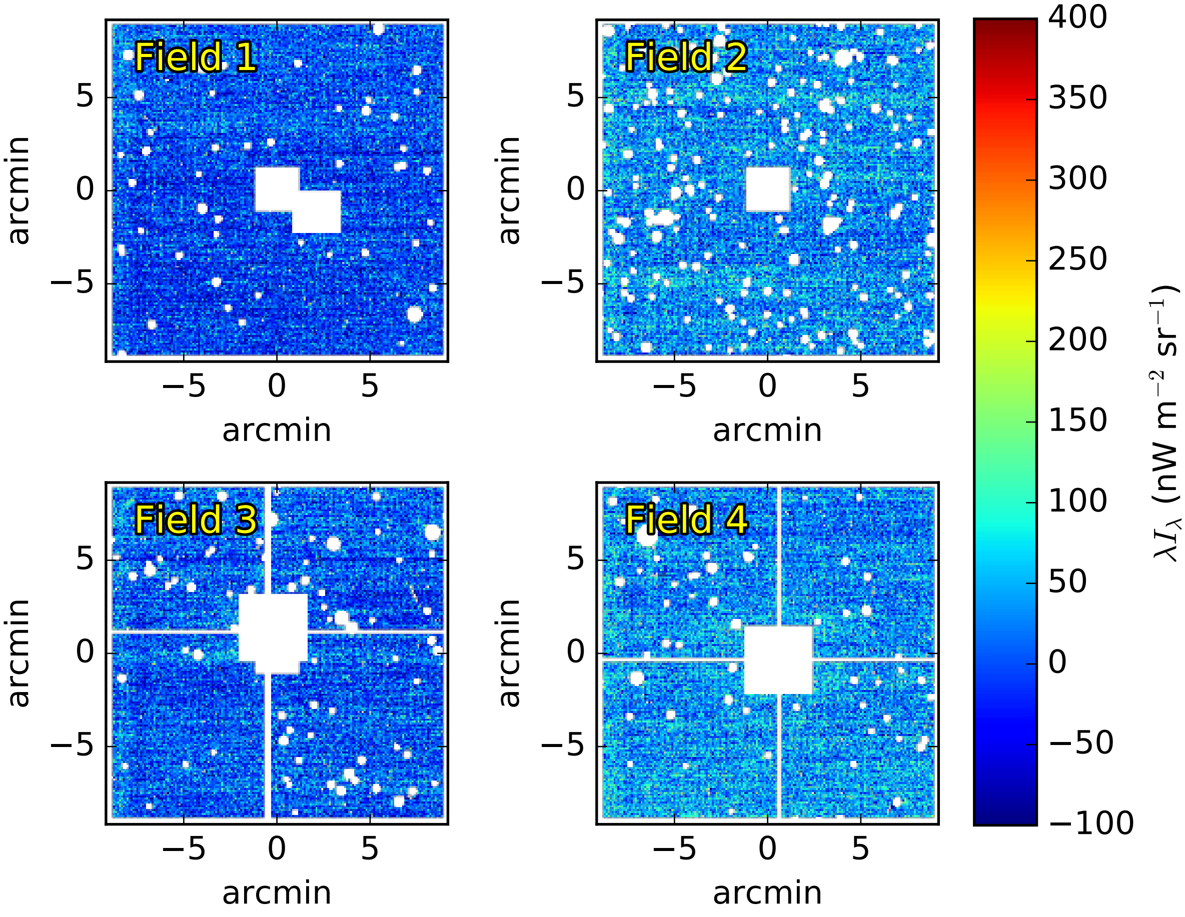

Supplementary Figure 3: **Masked, reduced images of the four science fields used in this investigation, calibrated to surface brightness.** Each panel shows one $10\,\mathrm{s}$ integration for each of the fields taken in rebinned 256×256 mode with the source and array masks applied. The mask effectively removes images of stars, planets, and optical and electronic pathologies in the images. The diffuse sky brightness λI_λ is computed from the mean of the unmasked pixels in these images. The mild horizontal striping is due to noise correlations in the output amplifier circuit, and are naturally present in any image of a sequentially read Si detector close to the noise floor. This effect causes the pixel variance to be a poor estimate of the absolute statistical error, though it remains a reasonable weight for averaging calculations where only changes in the exposure-to-exposure variance are relevant.

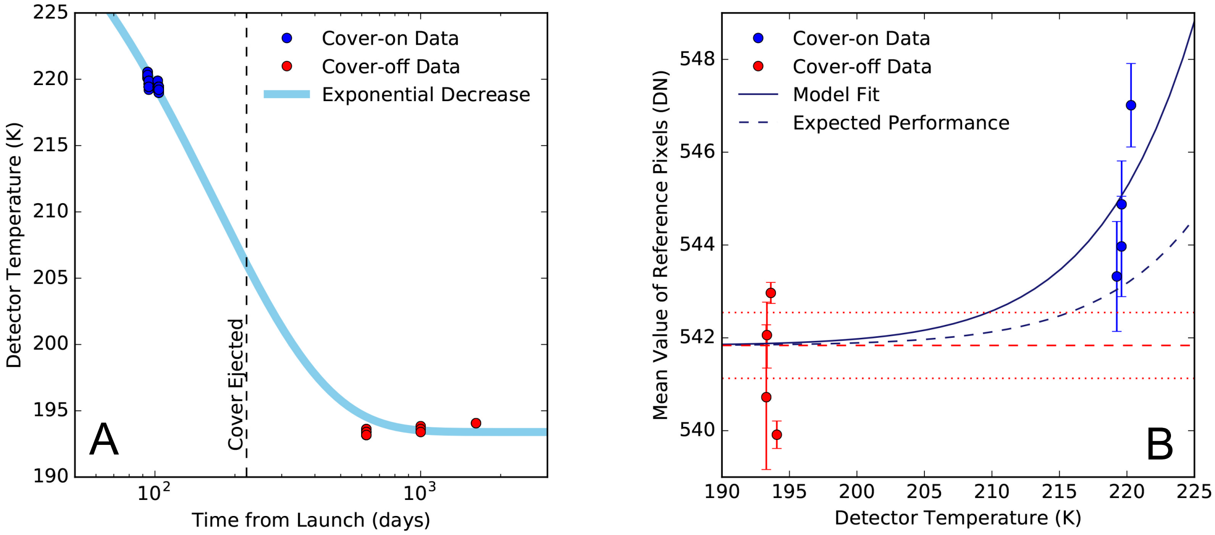

Supplementary Figure 4: **Temperature stability and reference pixel values over flight for both covered and uncovered data.** Measurements of the LORRI CCD temperature versus time for both dust cover on (blue points) and dust cover off (red points) are shown in panel A. We also indicate the time when the cover was ejected (dashed line) and the best-fitting exponential temperature decrease over the period of data collection. The plotted uncertainties correspond to the standard deviation of the individual measurements. Panel B shows the average value of the 256 dark reference pixels versus temperature over the flight. We indicate the mean cover-off reference pixel value (red dashed lines) and 1σ variation in the individual measurements (red dotted lines). Finally, a model for the dark current expected in these devices from the manufacturers specification (dashed blue line) and a free-amplitude fit of the same model (solid blue line) are shown. The dust-cover on data prefer an elevated dark current level above the baseline for this detector, but in either case the dark current is small at the 193 K operating temperature of the science observations. The residual reference fluctuations can be explained by temperature fluctuations in the electronics chain and bias generation (H. Weaver, private communication).

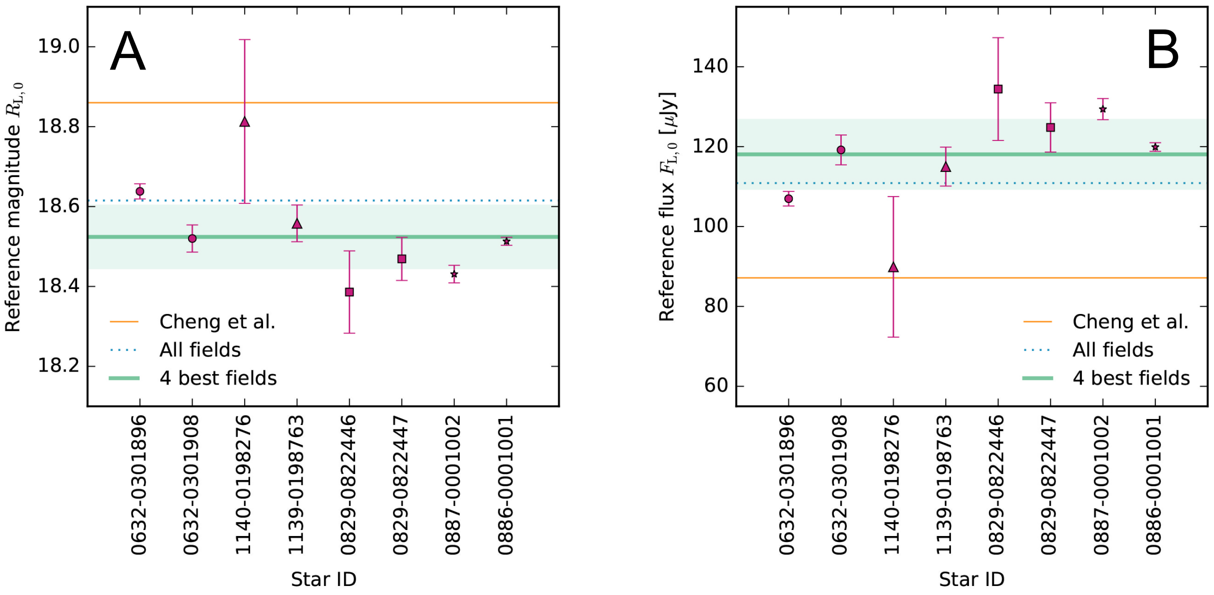

Supplementary Figure 5: **Summary of the photometric calibration in these LORRI data.** We plot the reference magnitude $R_{\mathrm{L},0}$ (panel A) and the reference flux $F_{\mathrm{L},0}$ (panel B). $R_{\mathrm{L},0}$ is calculated from aperture photometry as described in the text, and $F_{\mathrm{L},0}$ is calculated from $R_{\mathrm{L},0}$ using the PSF measurement. The points are computed using multiple measurements of two different stars in each of the four fields (circle, field 1; triangle, field 2; square, field 3; star, field 4). The average magnitude and flux of the stars in the four science fields are shown with the 1σ uncertainties, which we take as the best estimate of LORRI's photometric calibration. We compute $R_{\mathrm{L},0}$ and $F_{\mathrm{L},0}$ from all the available cover-off data, which matches the science field data within the errors. This shows that the reference magnitude and flux inferred from the four science fields are consistent with and representative of the entire sample. We also show the preliminary LORRI photometric calibration[3] converted to $R_{\mathrm{L},0}$-band; though our calibration is inconsistent with this measurement, it is based on a single observation of a crowded field with high photocurrents and no measurement uncertainty was specified. An improved photometric calibration of LORRI from the New Horizons team will be available in the near future.

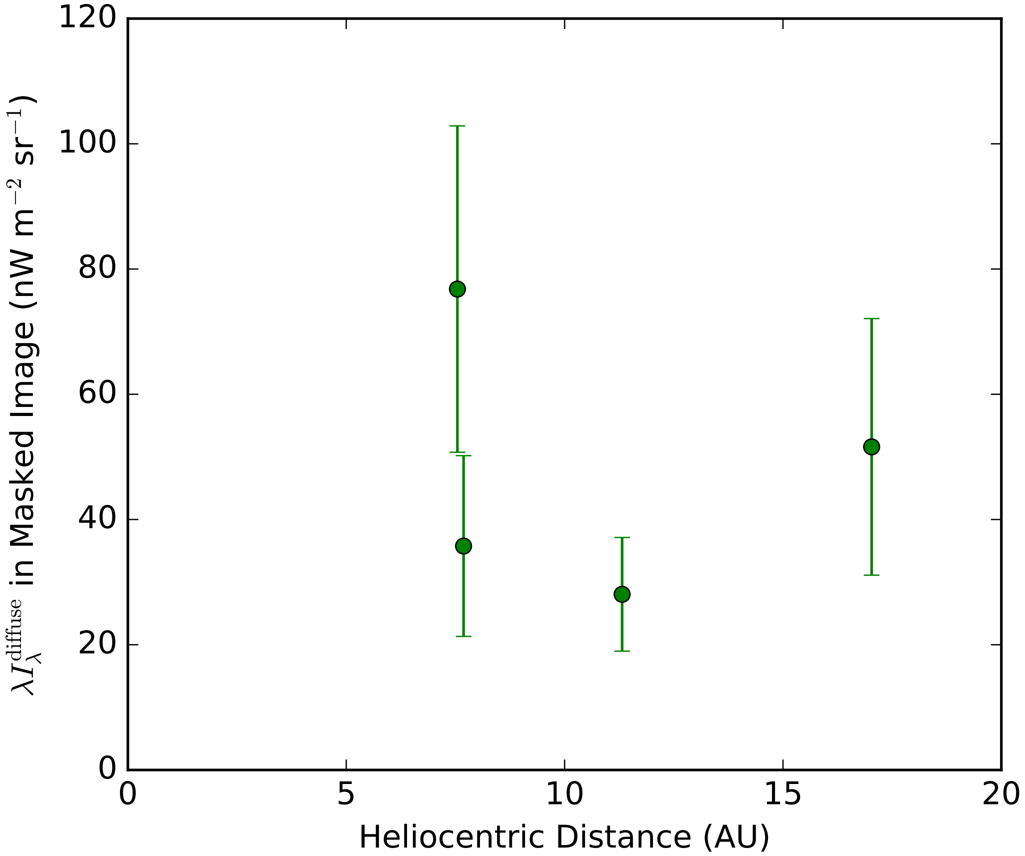

Supplementary Figure 6: **Measurement of $\lambda I_\lambda^{\rm diffuse}$ in the four science fields plotted as a function of heliocentric radius.** The points are computed from the variance-weighted mean of the individual $10\,\mathrm{s}$ exposures in each field, and the plotted uncertainties correspond to the standard deviation of the mean pixel value of each exposure on a given field, which should provide a reasonable estimate of the statistical noise in the data. These surface brightnesses still include emission from astrophysical foregrounds, which depend on field position in a complex way and must be estimated and removed to isolate the COB component. The first two points have been offset slightly in the horizontal direction to improve clarity.

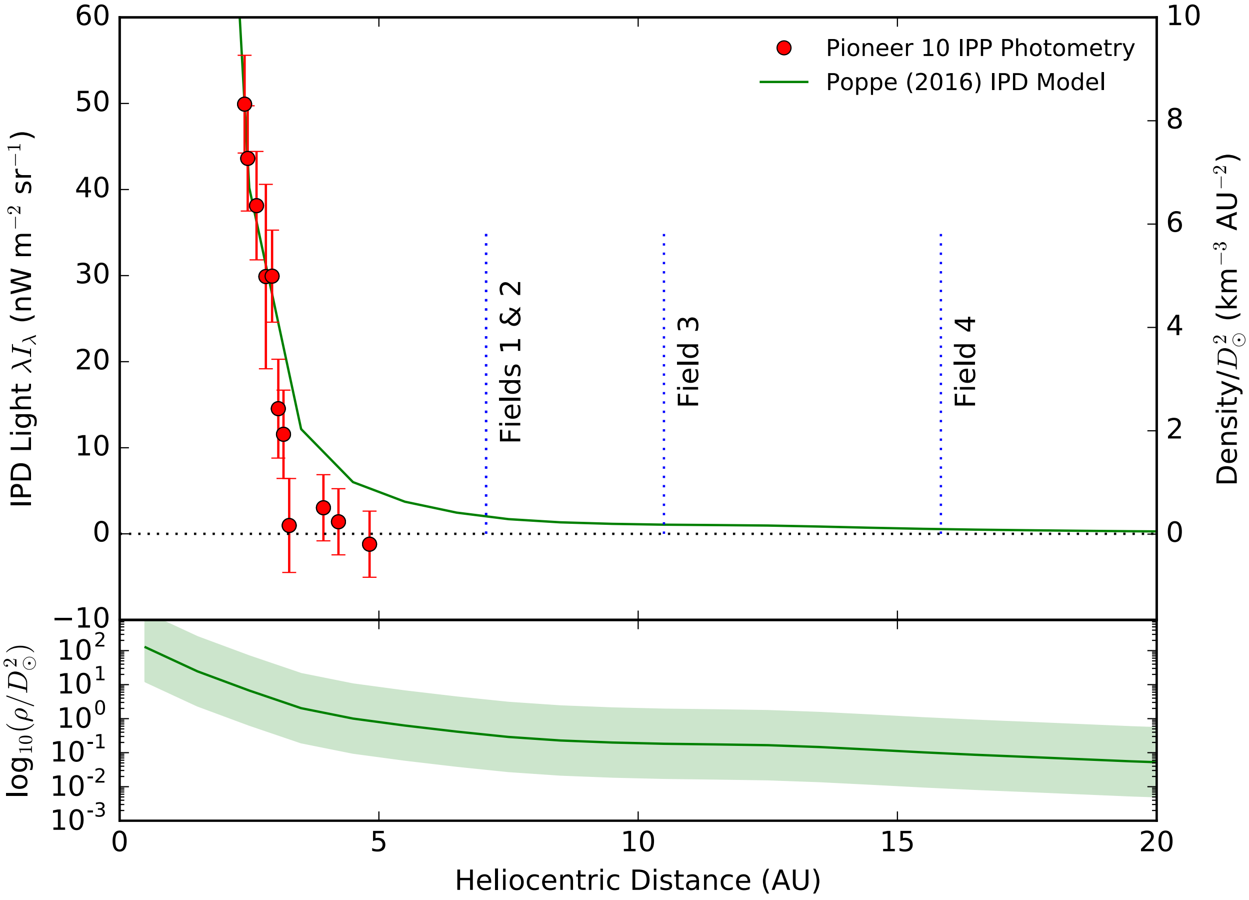

Supplementary Figure 7: **Surface brightness of the IPD and the $0.5-100\,\mu$m particle density per $R_\odot^2$ as a function of heliocentric distance.** We indicate the positions of the LORRI measurements by the labeled blue dashed lines. The red points show the photometric measurements from Pioneer 10 converted to nW m^{-2} sr^{-1} and referenced to $R_{\rm L}$-band[5]. For comparison, the value of $\lambda I_\lambda^{\rm ZL}$ near the earth is $\sim$ 1000 nW m^{-2} sr^{-1} at similar solar elongations, ecliptic latitudes, and wavelengths[6]. These data indicate a significant drop from 2 to 3.3 AU, and are consistent with zero beyond the asteroid belt. The green line shows the local IPD density predicted by the model of Poppe[7] divided by the square of the distance to the sun to account for the diminishing of intensity of sunlight with distance. The filled green region shows an estimate for the uncertainty in the density model. Assuming that the IPD light is sourced by particles close to the observer, and that the light scattering ability of these particles is independent of $R_\odot$, this model should exhibit the same behavior as the IPD light intensity. The model prediction follows the IPD light photometric measurements quite well. Although the absolute scaling between the dust flux and the IPD light is unknown, the general trend of a decrease from Earth followed by a flattening in the outer Solar system occurs in both measurements. Based on the IPD density model, we expect the IPD light to have a small and diminishing surface brightness in the plane of the outer solar system.

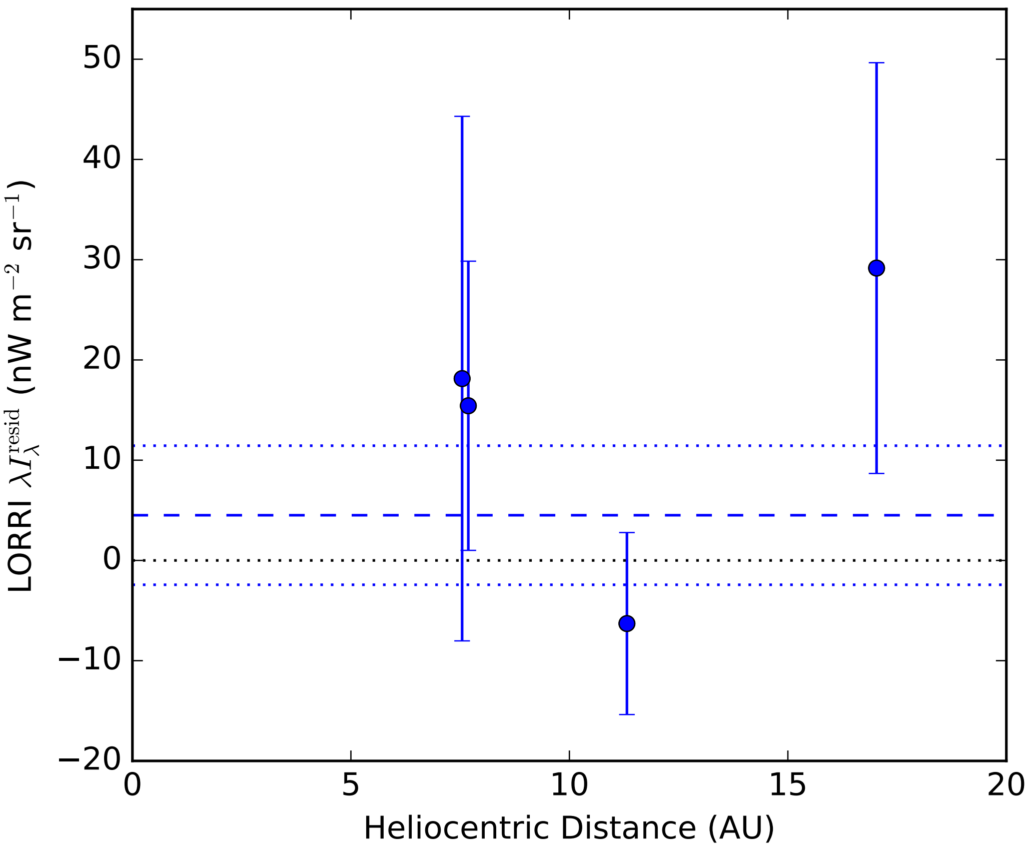

Supplementary Figure 8: **Residual surface brightness in the four LORRI science fields plotted as a function of heliocentric radius.** The points are computed from the variance-weighted mean of the individual 10 s foreground-subtracted exposures in each field, and the plotted uncertainties correspond to the standard deviation of the set of exposures, which should provide a reasonable estimate of the statistical noise in the data. We also plot the uncertainty-weighted mean and error of the four measurements (blue dashed and dotted lines, respectively). The unweighted mean lies at a larger value of 10.7 nW m^{-2} sr^{-1} in this plot. The first two points have been offset slightly in the horizontal direction to improve clarity.

Supplementary Table 1: *New Horizons* flight timeline.

Date	Nominal Target	Number of Records	Exposure Time per Record (s)	$R_\odot$ (AU)	$\theta_\odot$ (°)	$I_{100\mu m}$ (MJy sr^{-1})	Field Number
Launch 2006 January 19							
2006 April 23	Dark	120	10	1.8	-	-	D1
2006 April 24	Dark	59	10	1.8	-	-	D2
2006 May 2	Dark	120	10	1.9	-	-	D3
2006 May 3	Dark	60	10	1.9	-	-	D4
Cover Ejection 2006 August 29							
2006 August 31	Messier 7	4	1	3.4	142.6	38.3	R1
2006 September 21	$(\ell, b) = (13.1, 4.3)$	3	1	3.6	145.3	91.5	R2
2006 September 22	$(\ell, b) = (346.9, 31.5)$	6	1	3.7	177.9	7.6	R3
2006 September 24	$(\ell, b) = (13.1, 4.3)$	3	1	3.7	145.5	91.3	R4
2007 January 10	Callirrhoe	12	10	4.8	165.8	18.0	R5
Jupiter Closest Approach 2007 February 28							
2007 October 10	Makemake	10	10	7.6	85.5	2.4	R6
2007 October 10	Haumea	10	10	7.6	104.0	1.7	1
2007 October 10	Chariklo	10	10	7.6	94.2	4.4	2
2008 October 16	Neptune	3	10	11.3	104.8	3.6	3
2010 June 23	Neptune	3	10	17.0	95.4	2.8	4
2012 June 01	Pluto	5	10	23.3	167.8	147.7	R7
2013 July 01	Pluto	90	10	26.7	166.6	148.0	R8
2014 July 18	Pluto	48	10	29.9	165.7	147.0	R9
2014 July 20	Pluto	48	10	29.9	165.7	147.0	R10
End of Public Cruise Records 2014 July 20							

Supplementary Table 2: Foreground contributions to $\lambda I_\lambda^{\rm meas}$.

Field	λI_λ^*	$\lambda I_\lambda^{\rm diffuse}$	$\lambda I_\lambda^{\rm IPD}$	$\lambda I_\lambda^{\rm RS}$ from PSF wings	$\lambda I_\lambda^{\rm RS}$ from faint stars	$\lambda I_\lambda^{\rm DGL}$	$\lambda I_\lambda^{\rm resid}$
				(All nW m^{-2} sr^{-1})			
1	674	35.8	$< 2.4, 1\sigma$	0.02	12.7	7.7	15.4 ± 14.4
2	967	76.8	$< 2.4, 1\sigma$	0.13	7.8	50.8	18.1 ± 26.2
3	408	28.0	$< 2.4, 1\sigma$	0.01	6.7	27.6	-6.3 ± 9.1
4	242	51.6	$< 2.4, 1\sigma$	0.01	3.6	18.8	29.2 ± 20.5

Supplementary Table 3: Error budget for this measurement.

Error	Parameter Modification	Uncertainty in COB* (nW m^{-2} sr^{-1})
Statistical	$\lambda I_\lambda^{\rm COB} \pm 7.3$ nW m^{-2} sr^{-1}	± 7.3
Dark Current	$\lambda I_\lambda^{\rm inst} + 0.9$ nW m^{-2} sr^{-1}	-0.9
Optical Ghosts	$\lambda I_\lambda^{\rm inst} - 0.1$ nW m^{-2} sr^{-1}	–
Photometric Calibration Uncertainty	8% of $\lambda I_\lambda^{\rm diffuse}$	± 3.8
$\Omega_{\rm beam}$ Uncertainty	4% of $\lambda I_\lambda^{\rm diffuse}$	± 1.9
Aperture Photometry Loss	0.02% of calibration factor	–
Masking Bright Galaxies	$\lambda I_\lambda^{\rm diffuse} + 0.006$ nW m^{-2} sr^{-1}	–
IPD Light Uncertainty	$\lambda I_\lambda^{\rm IPD} + 2.4$ nW m^{-2} sr^{-1}	-2.6
0.25 mag USNO-B1 Photometry Uncertainty (per Source) on Source Mask	$\lambda I_\lambda^{\rm RS} \pm 0.1$ nW m^{-2} sr^{-1}	± 0.1
Sample Variance of Residual Faint Star Brightness	$\lambda I_\lambda^{\rm RS} \pm 0.6$ nW m^{-2} sr^{-1}	± 0.7
DGL-$100\,\mu$m Model Uncertainties	$\lambda I_\lambda^{\rm DGL}\{-8.7, +8.2\}$ nW m^{-2} sr^{-1}	$\{-9.1, +8.6\}$
Galactic Extinction	$(1.05 \pm 0.01) \cdot \lambda I_\lambda^{\rm residual}$	–

* '–' denotes a negligible error.

Supplementary References

1. Bock, J. *et al.* The Cosmic Infrared Background Experiment (CIBER): The Wide-field Imagers. *The Astrophysical Journal Supplement* **207**, 32 (2013).

2. Morgan, F. *et al.* Calibration of the New Horizons Long-Range Reconnaissance Imager. In *Astrobiology and Planetary Missions* (Eds. Hoover, R. B. et al.) 421-432 (2005).

3. Cheng, A. F. *et al.* Long-Range Reconnaissance Imager on New Horizons. *Space Science Reviews* **140**, 189–215 (2008).

4. Owen Jr, W. M., Dumont, P. J. & Jackman, C. D. Optical navigation preparations for new horizons pluto flyby. In *Astrobiology and Planetary Missions: 23rd International Symposium for Space Flight Dynamics, Pasadena, CA* 421-432 (2012).

5. Hanner, M. S., Weinberg, J. L., DeShields, L. M., II, Green, B. A. & Toller, G. N. Zodiacal light and the asteroid belt: The view from Pioneer 10. *Journal of Geophysical Research* **79**, 3671 (1974).

6. Leinert, C. *et al.* The 1997 reference of diffuse night sky brightness. *Astronomy and Astrophysics Supplement* **127**, 1–99 (1998).

7. Poppe, A. R. An improved model for interplanetary dust fluxes in the outer Solar System. *Icarus* **264**, 369–386 (2016).
